# Quality of Life Among Family Caregivers of Disabled Children in Saudi Arabia

**DOI:** 10.7759/cureus.41320

**Published:** 2023-07-03

**Authors:** Ghofran H Sulaimani, Shady Kamel, Ghadi Alotaibi, Nada Telmesani

**Affiliations:** 1 Preventive Medicine and Public Health Department, Ministry of Health, Riyadh, SAU; 2 Saudi Field Epidemiology Training Program, Ministry of Health, Riyadh, SAU; 3 Neurological Service of Physical Medicine and Rehabilitation, Nantes Université, Nantes, FRA

**Keywords:** saudi arabia, short-form 36, children with disabilities, caregivers, quality of life

## Abstract

Background: The physical and mental health of parents can be adversely affected by their child’s disability, leading to a potential decline in their overall Quality of Life (QoL). This research aimed to determine the QoL of family caregivers and compare this based on sociodemographic factors and child characteristics.

Materials and methods: An analytical cross-sectional design was employed, and data were collected from multiple rehabilitation centers for children with special needs and learning disorders in the Kingdom of Saudi Arabia. The study sample comprised 95 family caregivers who completed a self-structured questionnaire. The questionnaire included sections on sociodemographic characteristics, child characteristics, and caregivers’ QoL assessed using the RAND 36-Item Short Form Health Survey (SF-36). The collected data were analyzed using the IBM Corp. Released 2022. IBM SPSS Statistics for Windows, Version 29.0. Armonk, NY: IBM Corp, employing descriptive statistics and multivariate linear regression analysis.

Results: Our findings revealed that the overall mean QoL score among caregivers was 57, ranging from 12 to 94. There were no statistically significant differences in QoL scores based on caregivers’ age, gender, occupational status, or income. However, further analysis indicated significant associations between certain factors and specific domains of QoL. Specifically, caregiver education was found to be associated with role limitations due to emotional problems, while relationships with disabled children were linked to emotional well-being. The severity of the child’s disability and the presence of another child with a disability in the family were associated with the bodily pain domain. Additionally, the presence of another child with a disability had an impact on perceived change in the health domain.

Conclusions: The QoL of family caregivers varied, highlighting the significant challenges faced by certain individuals. Factors such as education level, the relationship with the child, the severity of the disability, and the presence of multiple disabled children in the family were identified as influencing caregivers’ QoL. These findings emphasize the importance of developing targeted interventions that can address emotional well-being and fatigue management while promoting a supportive society.

## Introduction

According to the Centers for Disease Control and Prevention (CDC), disability can be defined as “any impairment of the body or mind that makes it more difficult for the person with the condition to do certain activities (activity limitation) and interact with the world around them (participation restrictions)” [1].

Disability in developing and developed countries is an important public health problem. In the United States, about one in four people (26%) in the community suffer from a disability [2]. Globally, the number of individuals living with disabilities surpasses one billion, which accounts for 15% of the global population [3]. The World Health Survey estimated that approximately 785 million people over the age of 15 are living with a disability, while the global burden of disease is estimated to be approximately 975 million. The Global Burden of Disease is the only measure of childhood disability (ages 0-14), with an estimated 13 million of an estimated 95 million (5.1%) children thought to be severely disabled. Changing demographics and an increase in chronic diseases mean that disability will become an even greater problem over the next few years [3]. In 2017, the General Authority for Statistics (GaStat) estimated that 7.1% of the total population of Saudi Arabia had a disability (mild, severe, or extreme). Males make up 52.2% of the population, and females make up 47.8%. Geographically, the survey results indicate that the Riyadh region has the highest disability rate (25.13%) compared to other Saudi Arabian regions [4].

The population of children with disabilities is diverse, including children with genetic conditions, brain or spinal cord injuries, nutritional deficiencies, and infections resulting in long-term cognitive, mobility, visual, auditory, and behavioral impairments. This is in addition to environmental toxin exposure and those who experience anxiety or depression due to stressful life events [5]. The disability of a child has a detrimental effect on the physical and psychological well-being of their parents, which can potentially lead to a diminished overall QoL. The World Health Organization defined QoL as “an individual’s perception of their position in life in the context of the value systems and culture where they live and concerning their purposes, standards, concerns, and expectations. It can be a broad-ranging concept influenced in a complicated way by the person’s physical well-being, mental state, individual opinions, social connections, and their correlation to notable specifications of their environment” [6]. Coping with the enduring challenges of raising a child with a lifelong disability can be demanding, involving physical, mental, and financial hardships. As a result, children have become increasingly reliant on their parents, which poses new obstacles in terms of providing care for a child who has a chronic illness [7]. Furthermore, caregivers encounter various forms of functional limitations that necessitate additional effort and specialized attention, particularly when compared to children of a similar age and gender [8]. Additionally, the emotional distress and worries that parents may experience in the future, as well as the social stigma associated with a child who has developmental delays, can have a negative impact on caregivers’ QoL [9].

Several studies that compared the QoL of mothers with and without disabled children found that the QoL of mothers with disabled children was significantly lower [10-12]. A cross-sectional study conducted in the Kingdom at the time of the COVID-19 lockdown revealed that primary caregivers of disabled children experienced decreased QoL scores in social, environmental, and leisure activities. Furthermore, the mothers of children with disabilities have reported issues that emerged at the time of the COVID-19 lockdown, highlighting concerns such as a decline in the health status of their children and their limited availability of essential medical supplies [12]. In another study conducted in 2020, it was found that 2/3 of caregivers of children with attention-deficit/hyperactivity disorder (ADHD) had poor QoL. Notably, the psychological aspect had the greatest negative impact, while the environmental aspect had the least influence [13]. One study revealed the difficulty experienced by caregivers in coping with the various stresses associated with caring for their chronically ill children due to the limited resources and facilities available for their children [14]. Given the cultural norms, women in Eastern countries are expected to care for the house and all members of their family who live inside. This may include grandparents [15]. Therefore, mothers carry the primary responsibility, as they are entrusted with ensuring their child’s adherence to treatment, medical appointments, and dietary requirements. In addition to taking care of their sick child, mothers also have to take care of other family members every day [14,16].

Consequently, it is crucial to study the impact of having a child with a disability on QoL for caregivers; it is important to ensure that their well-being is reflected in their children. Only a few previously published studies in Saudi Arabia have addressed this issue. Hence, this research was conducted to investigate the QoL of family caregivers of disabled children in Saudi Arabia. According to the Convention on the Rights of Persons with Disabilities (CRPD), children with disabilities “include those who have long-term physical, mental, intellectual, or sensory impairments that, in interaction with various barriers, may hinder their full and effective participation in society on an equal basis” [5]. In 2016, UNICEF and the Washington Group on Disability Statistics showed that 1 in 10 of all children worldwide have disabilities [5]. Based on a recent report by Saudi Arabia’s General Authority for Statistics (GAStat), the prevalence rate of disability among those aged 5 and over in the total Saudi population is 78 per 1000 [4].

The assistance required by this population varies depending on each disability. It is well documented that parent caregivers suffer from physical, emotional, social, and financial burdens over the years of caring for their disabled child. As part of Saudi Arabia’s Vision 2030, several Vision Realization Programs (VPRs), such as the Quality-of-Life Program, were launched in 2018 [17]. These initiatives have helped focus efforts on all aspects of QoL to ensure high living standards for residents and visitors alike. Along these lines, investigating the QoL of caregivers who care for disabled children is of the utmost importance. This area of concern requires a comprehensive exploration, specifically within the context of Saudi Arabia. The aims of our current study were two-fold: first, to determine the QoL among the family caregivers of disabled children in Saudi Arabia; second, to compare QoL based on sociodemographic factors and child characteristics.

## Materials and methods

Study design

An analytical cross-sectional design was utilized in this study, which was conducted from 1 December 2022 to 31 January 2023.

Study setting

This study was conducted in multiple rehabilitation centers for children with special needs and learning disorders in the Kingdom, including King Fahad Medical City (KFMC) and Alawael Center for Rehabilitation in the Riyadh region, as well as Sinad City for Special Education in the Makkah region. The Ministry of Health (MOH) is primarily responsible for providing health services in Saudi Arabia. Over the past two decades, the Ministry of Health has built several rehabilitation services for disabled individuals and other citizens of the country. Most of these programs provide services for physical therapy, occupational therapy, speech therapy, and hearing therapeutic interventions, as well as services for prosthetics and orthotics. These services have been integrated into the existing modern and sophisticated healthcare service system and infrastructure [18].

Study subjects

The subjects for this study were selected using a convenient non-probability sampling technique. The study sample comprised 117 responses from family caregivers of children with various types of disabilities. The inclusion criteria were a child's age of fewer than 18 years, agreement to participate in the study, both genders, Saudi nationality, literacy, and a social media account for the caregiver. Conversely, the exclusion criteria were non-family caregiver of a child with a disability, non-Saudi nationality, child's age of more than 18 years, incomplete responses, no social media account for the caregiver, and refusal to participate in the study. Therefore, 95 respondents were eligible for this study.

Sample size

The targeted sample size was determined using an open-source calculator (Raosoft website). In accordance with data from the General Authority for Statistics of the Kingdom of Saudi Arabia, the prevalence rate was established at 7.78%, with a confidence interval of 95% and a margin of error set at 5%. Based on these parameters, the estimated total sample size was calculated to be 110 responses.

Data collection procedures and tool

Data were collected through the distribution of a link to an online, self-structured, three-part questionnaire to caregivers in various regions. An online survey system was used to generate a questionnaire in Arabic via Google Forms. The generated link was conveniently shared on social media platforms (i.e., WhatsApp, Twitter, and Telegram). The study's objective was clearly explained within the questionnaire interface. The questionnaire itself encompassed three sections: the sociodemographic characteristics of the caregiver, the characteristics of the child, and an assessment of the caregivers' QoL.

The first section of the questionnaire included eight questions pertaining to the personal information of the caregiver. These questions encompassed aspects such as age, nationality, gender, level of education, marital status, occupation, perceived economic status, and relationship with the child. The level of education was dichotomously classified as “educated” or “non-educated”. The designation of an individual as “uneducated” was based on the absence of formal schooling or the completion of education only up to the primary school level. Conversely, an individual was recognized as “educated” if they had acquired education beyond the primary school level [19,20]. Marital status was categorized as married, divorced, or widowed. The occupation was classified as either “employed” or “unemployed”. Perceived economic status was assessed based on the reported average monthly wage according to the General Authority for Statistics (GASTAT) 2018 data [21]. This was further subdivided into three categories: less than 5000 Saudi Riyals per month, between 5000 and 15,000 Saudi Riyals per month, and exceeding 15,000 Saudi Riyals per month. Moving on to the second section, which focused on the characteristics of the child. This section encompassed inquiries regarding the child’s gender, age, birth order, number of siblings, types of disability, the severity of disability, and whether there was another child with disabilities in the family. The third section of the questionnaire focused on an assessment of the caregivers’ QoL, utilizing the Arabic version of the RAND 36-Item Short Form Health Survey (SF-36). The SF-36 is a validated set of generic, coherent, and easily administered QoL measures. It comprises 36 items and can be measured using the following eight health aspects: physical functioning, bodily pain, role limitations due to physical health problems, role limitations due to personal or emotional problems, emotional well-being, social functioning, energy/fatigue, and general health perceptions.

Ethical consideration

Ethical approval was obtained from the Institutional Review Board of King Fahad Medical City in Riyadh, Saudi Arabia (IRB Log No. 22-550E) on 27 November 2022. The survey included a consent question to ensure that the respondents agreed to participate in the study. The survey description provided complete information about the study and the contact details of the principal investigator. The confidentiality of the data collected was strictly maintained and not disclosed for any purposes other than the study.

Statistical analysis

The software IBM Corp. Released 2022. IBM SPSS Statistics for Windows, Version 29.0. Armonk, NY: IBM Corp was used for data entry and statistical analysis. Descriptive statistics were reported for both sociodemographic and child characteristics. Continuous variables were expressed as the mean ± standard deviation (SD), and range, while categorical variables were presented as frequencies and percentages. The one-way analysis of variance (ANOVA) test was employed for the comparison of continuous variables among three or more groups, whereas the Student’s t-test was used to compare the means within two groups. A significance level of p≤ 0.05 was considered for all statistical tests. The QoL among family caregivers was assessed using the SF-36 questionnaire, which measured eight health aspects. Each item was scored on a scale of 0 to 100. Considering a score of 100 as favorable indicates a good health state. The mean and standard deviations were calculated from the recorded scores for each health aspect. Items with missing data were not taken into account when calculating the scale scores. To identify significant differences in the QoL domains among family caregivers, a multivariate linear regression analysis was performed.

## Results

Sociodemographic characteristics

Table 1 displays the sociodemographic characteristics of the caregivers. A total of 48 respondents (56.5%) were aged between 36 and 45 years. Most caregivers were mothers, accounting for 48 individuals (51.6%). Furthermore, most participants, comprising 78 individuals (83%), were married and lived together. In terms of education, 84 caregivers (88.4%) were educated. Regarding income, a slight majority of caregivers, 51 individuals (53.7%), reported a moderate income level ranging between 5000 and 15,000 Saudi Riyals per month.

**Table 1:** Sociodemographic characteristics of the caregivers.

Characteristic		Count	N %
Caregiver’s Age	26–35	21	24.7%
	36–45	48	56.5%
	46–55	9	10.6%
	56–65	7	8.2%
Gender	Female	56	58.9%
	Male	39	41.1%
Carer’s Education	Educated	3	3.4%
	Not educated	6	6.7%
	3.00	25	28.1%
	4.00	8	9.0%
	5.00	40	44.9%
	6.00	5	5.6%
	8.00	2	2.2%
Marital Status	married	78	83.0%
	Not married	16	17.0%
Carer’s Occupation	Employed	48	50.5%
	Not Employed	47	49.5%
Relation	Father	37	39.8%
	Mother	48	51.6%
	Brother	2	2.2%
	Sister	0	0.0%
	other	6	6.5%

Child characteristics

The characteristics of the child participating in the study are presented in Table 2. Most of the children, 40 (44%), were aged between 11 and 18 years. In terms of gender distribution, approximately 55.8% of the children were male. Moreover, most of the children, specifically 28 individuals (57.1%), exhibited a physical disability. It is noteworthy that a considerable number of children, 42 individuals (44.2%), presented with a moderate level of disability.

**Table 2:** The characteristics of children with disabilities.

Characteristic			Count	N %
Child’s Age		1–5	14	15.6%
		6–10	36	40.0%
		11–18	40	44.4%
Number of Children		two or less	31	35.6%
		three or more	56	64.4%
Order of Child		first	27	28.4%
		second	16	16.8%
		third or more	52	54.7%
Child’s Gender		Female	42	44.2%
		Male	53	55.8%
Previous Child Disability	yes		8	8.4%
	no		87	91.6%
Severe Disability	mild		17	17.9%
	moderate		42	44.2%
	severe		36	37.9%
Type of Disability	physical		28	57.1%
	intellectual		0	0.0%
	visual		0	0.0%
	hearing		0	0.0%
	speech		3	6.1%
	growth failure		1	2.0%
	learning		1	2.0%
	sensory		0	0.0%
	autism		3	6.1%
	ADHD		3	6.1%
	down syndrome		5	10.2%
	cerebral palsy		5	10.2%
	epilepsy		0	0.0%
	other		0	0.0%

Quality of life of the caregivers

The primary objective of this study was to determine the QoL among the family caregivers of disabled children in Saudi Arabia. The Mean ± SD of the QoL score among the caregivers was found to be 57 ± 17, with a maximum value of 94 and a minimum value of 12.

In our study, we assessed the QoL of family caregivers by measuring the eight domains of health separately. Since the average score obtained for all eight domains did not yield statistically significant results, we conducted multiple linear regression analyses for each of the eight health domains to investigate the relationship between the QoL of the caregivers and their sociodemographic and child characteristics.

**Figure 1:**
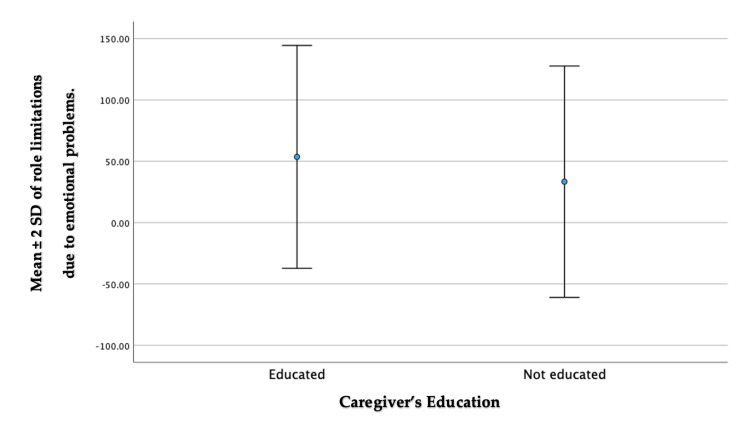
Whisker plot depicting a comparison between the educated and non-educated groups in the role limitations due to emotional problems domain.

This analysis revealed a statistically significant association between the items assessing role limitations due to emotional problems and the caregiver’s education (p = 0.027). Among the caregivers who were educated, the mean score ± SD was 53.57 ± 45.41. By contrast, the non-educated caregiver group had a lower mean score of 33.33 ± 47.14 for the same domain (Figure 1).

**Figure 2:**
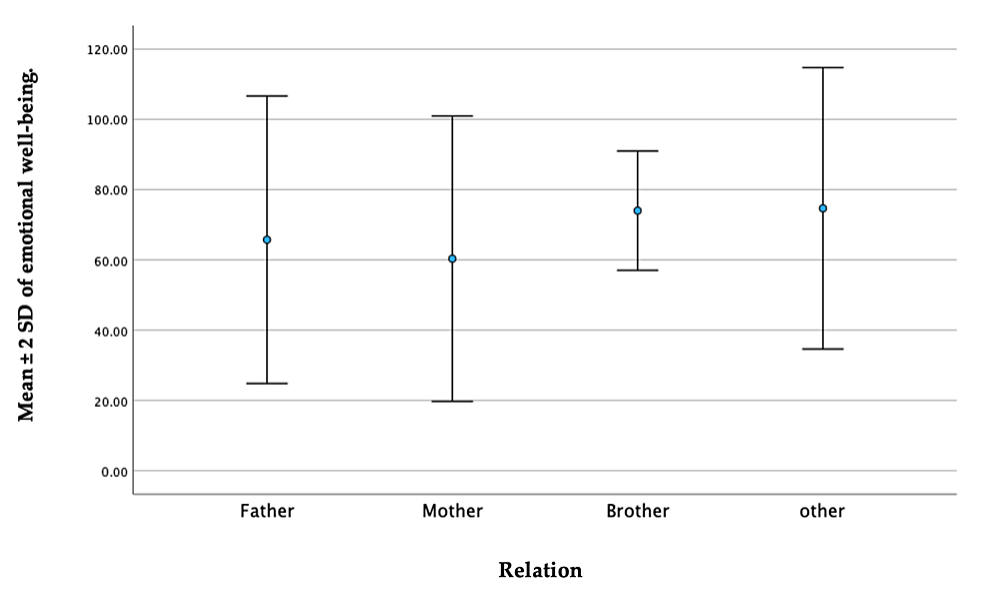
A whisker plot depicting a comparison between the caregiver's relationship to a disabled child in the emotional well-being domain

The statistical analysis demonstrated a significant relationship between the items assessing emotional well-being and the caregiver’s relationship with the disabled child (p = 0.023). Notably, there were differences observed in the emotional well-being scores between mothers and brothers who served as caregivers. Mothers, on average, had a slightly lower emotional well-being score, with a mean ± SD of 60.33 ± 20.31. On the other hand, brothers exhibited a higher average emotional well-being score with a mean ± SD of 74.00 ± 8.49 (Figure 2).

**Figure 3:**
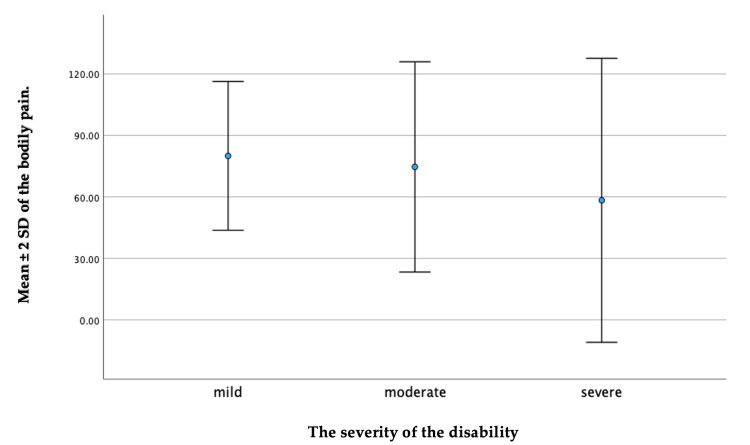
A whisker plot depicting a comparison between the severity of disability in the bodily pain domain.

**Figure 4:**
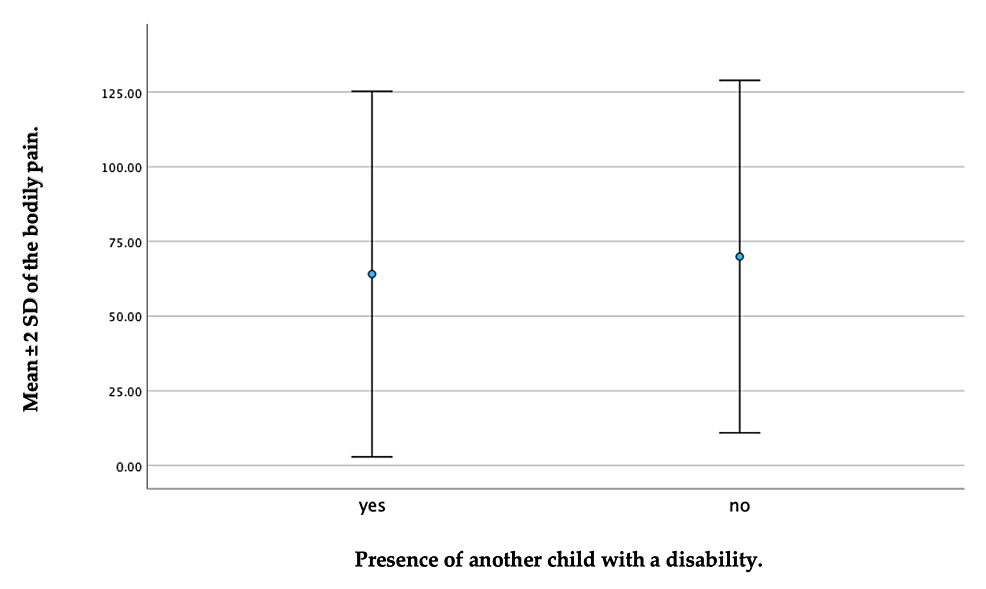
A whisker plot depicting a comparison between caregivers who had a previous child with a disability or not in the bodily pain domain.

It has been shown that the severity of a child’s disability (p = 0.047) and the presence of another child with a disability (p = 0.027) are linked to a lower QoL scale in the bodily pain domain. Regarding the severity of the disability, children with a mild disability had a higher mean ± SD of 80.00 ± 18.14 on the QoL scale. This could be compared to the caregivers of children with severe disabilities, who reported the lowest average, with a mean ± SD of 58.33 ± 34.63 (Figure 3). Furthermore, caregivers who had a previous child with a disability had a mean ± SD of 64.06 ± 30.59 on the QoL scale in relation to bodily pain. By comparison, caregivers without a previous child with a disability had a slightly higher average, with a mean ± SD of 69.91 ± 29.50 (Figure 4).

**Figure 5:**
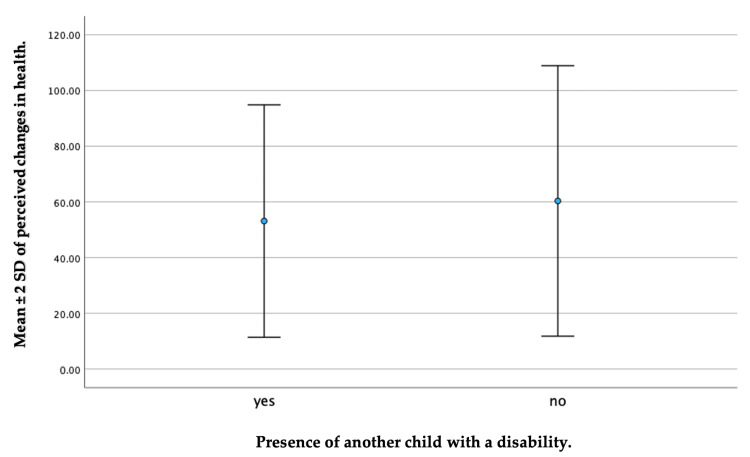
A whisker plot depicting a comparison between caregivers who had a previous child with a disability or not in the perceived change in the health domain.

In addition, there was a significant association between the item assessing perceived changes in health and the presence of another child with a disability (p = 0.027). For caregivers who had a previous child with a disability, the mean ± SD on the QoL scale in relation to a perceived change in health was 53.13 ± 41.72. By contrast, caregivers without a previous child with a disability had a more favorable health change, with a mean ± SD of 60.34 ± 48.56 (Figure 5).

Correlation between all domains

Table 3 demonstrates the correlation between all domains of QoL. A significant and strong positive correlation could be observed between role limitations due to physical health problems and role limitations due to emotional problems, with a correlation coefficient (r = 0.682). Additionally, the energy/fatigue domain exhibited significant and strong positive correlations with emotional well-being (r = 0.725), social functioning (r = 0.627), and bodily pain (r = 0.602).

**Table 3:** Correlation between eight domains of SF-36. The table shows the values of Pearson’s correlation. ** significance at P ≤ 0.01

Domains	Physical functioning	Role limitations due to physical health	Role limitations due to emotional problems	Energy/fatigue	Emotional well-being	Social functioning	Pain	General health
Physical functioning								
Role limitations due to physical health	.535**							
Role limitations due to emotional problems	.370**	.682**						
Energy/fatigue	.124	.455**	.413**					
Emotion-al well-being	.103	.287**	.430**	.725**				
Social functioning	.171	.384**	.476**	.627**	.633**			
Pain	.212	.402**	.262	.602**	.480**	.597**		
General health	.166	.339**	.245	.467**	.520**	.465**	.582**	

## Discussion

QoL has emerged as a crucial indicator in public health due to its holistic reflection of an individual’s physical, mental, and social well-being [7]. Caring for a disabled child presents an array of challenges that can profoundly impact the QoL of a caregiver [14,16]. Numerous studies have investigated the variables affecting the well-being of caregivers, realizing that their QoL is intrinsically linked to the child’s overall development and the support structures available to that family [9,22]. In this study, the primary objective was to assess the QoL of family caregivers who were taking care of disabled children in Saudi Arabia. To achieve this, QoL scores were measured using the Arabic version of the SF-36 to enable a comprehensive assessment of the various dimensions that contribute to caregivers’ QoL. The SF-36 questionnaire utilizes a scoring range of 0 to 100 to assess various domains of health and functioning. These scores represent the percentage of the total possible score achieved. Higher scores indicate a more favorable health state. Caregivers who obtained higher scores on the SF-36 had better QoL outcomes, suggesting that they experienced improved well-being and functioning in the assessed areas.

The mean QoL score obtained was 57, which suggests that, on average, the family caregivers in our sample experienced a moderate level of QoL. It is important to note that the QoL scores encompass various aspects of caregivers’ lives, including their physical, emotional, social, and financial well-being. The spread of scores, ranging from 12 to 94, indicates a wide variation in QoL among caregivers; while some caregivers reported relatively high levels of QoL, others clearly faced significant challenges in maintaining their well-being. To provide a comprehensive understanding of the QoL of family caregivers, it is crucial to consider the sociodemographic factors and characteristics of the child that may influence their well-being. Therefore, this study’s secondary objectives were to compare QoL scores based on sociodemographic factors and child characteristics.

With regard to the sociodemographic characteristics of caregivers, we found no statistically significant differences between caregivers’ age and gender and their QoL scores. These results contrast with previous studies in the literature, which report lower QoL scores among caregivers in older age groups where being female is associated with reduced QoL scores [23-26]. Findings from a study conducted in Saudi Arabia revealed that caregivers aged over 50 exhibited the lowest scores in various domains of the SF-36 questionnaire, such as physical functioning, role limitations due to emotional problems, social functioning, bodily pain, and general health perceptions. By contrast, caregivers between the ages of 41 and 50 had the lowest scores in this domain of perceived change in health [27]. This discrepancy observed in our study could be attributed to individual variations within age groups, including factors such as the presence of pre-existing health conditions, personal coping mechanisms, and social support networks. These individual differences could contribute to the lack of significant differences observed between age groups in our study.

In relation to education level, our results indicated that educated caregivers experienced higher scores in role limitations due to the emotional problems domain compared to their non-educated counterparts. This suggests that caregivers with lower levels of education perceive more challenges when managing their emotional well-being and fulfilling their caregiving roles. There could be several possible explanations for this finding. Caregivers with lower levels of education may have limited access to information, resources, and support systems that can help them effectively cope with emotional challenges. Additionally, they may have fewer opportunities for personal growth and self-development, which could contribute to a higher sense of role limitations in the emotional domain. Furthermore, lower levels of education may be associated with socioeconomic disadvantages, such as limited employment opportunities or financial constraints. These stressors could exacerbate emotional difficulties and increase the perceived impact on caregiving responsibilities.

In terms of caregiver occupational status and income, it is noteworthy that we failed to find a significant relationship. This was consistent with previous research that also reported no correlation between parents’ income and their QoL [28-30]. However, these findings contradict other studies that found low-income and unemployed caregivers to have considerably poorer QoL [11,31,32]. The role of this work is, therefore, important in shaping parenting behaviors. When working hours, workload, and complexities arise, parents may encounter difficulties in meeting the demands of their dual roles as employees and parents [13]. This could result in conflicts between work and family responsibilities, leading to psychological issues caused by emotional exhaustion [33,34]. Additionally, the parents of disabled children often face additional healthcare costs and the need for specialized facilities, further increasing their financial burden [10,35,36]. A study found a significant association between the availability of health insurance and overall QoL, which could be attributed to the improved accessibility of health services [31]. These disparities in the relationship between caregiver QoL, occupational status, and income may be attributed to the comprehensive care services provided by the Kingdom of Saudi Arabia. The Kingdom offers a range of services to individuals with disabilities, aiming to support their overall well-being and promote their integration into society. These services encompass medical, social, psychological, educational, and professional assistance, which are all geared toward maximizing functional effectiveness and facilitating their inclusion in a normal environment and social life. Additionally, the Kingdom provides financial support and assistive devices to individuals with disabilities to help them meet their daily living expenses and cope with the additional costs associated with their disability [37].

In addition, the caregiver’s relationship with the disabled child plays a critical role in shaping their emotional well-being [13,29,38]. Specifically, our study revealed that mothers, on average, had slightly lower scores in emotional well-being compared to other family members. This aligns with previous research that has also reported worse QoL in caregiver mothers compared to fathers [33,39-41]. For instance, several studies focusing on the caregivers of children with ADHD found that mothers experienced higher levels of emotional exhaustion, depersonalization, and decreased personal accomplishment [42-44]. One possible explanation for this is that mothers often assume the primary caregiving responsibilities and face additional stressors and demands in their roles [10]. These challenges include managing daily care tasks, coordinating medical appointments, and providing emotional support to the disabled child. Along with providing care, mothers are also responsible for attending to the needs of siblings and the rest of the family [14]. The cumulative effect of these responsibilities may contribute to a higher perceived burden and emotional strain among mothers, resulting in lower emotional well-being scores [44-46]. Our outcome is consistent with previous research, which has discovered that the mothers of children with chronic kidney disease achieved significantly lower scores in total QoL, as well as in physical health, mental health, and general well-being, in comparison to their fathers [47].

In line with our investigation, several previous studies have examined the influence of the child’s characteristics on the QoL of caregivers [22,31,48]. For instance, a study conducted in Arar City revealed that parents who had a first-born child diagnosed with autism were found to have a fivefold higher likelihood of experiencing poor QoL compared to parents whose child with autism was born third or later in the birth order [11]. Additionally, another study reported that parental health-related quality of life (HRQOL) and family functioning scores were lower for parents of male children with disabilities than for those of female children with disabilities [41]. This finding contrasts with previous studies, which observed that the parents of female children with autism experienced significantly lower QoL [38]. However, the current study has found no statistical differences in the QoL of caregivers based on the age, gender, or birth order of their child. Similarly, a study conducted in Khartoum did not find any correlation between the age of children with cerebral palsy and the QoL experienced by their parents [31]. It is important to consider that each study is conducted on a unique sample, and variations in the characteristics of caregivers and disabled children within the sample can influence the study’s outcomes. Our study sample may have had a more diverse range of ages and birth orders among disabled children, leading to a more balanced distribution and ultimately resulting in no statistically significant differences in QoL.

In this article, we also sought to explore the relationship between the type of disability and caregivers’ QoL [33,34]. We included a range of disability types, such as physical disabilities, sensory disabilities, intellectual disabilities, developmental disabilities, visual impairments, hearing impairments, and communication disabilities. However, our investigation did not reveal a significant association between the type of disability and caregivers’ QoL. It is important to acknowledge that the absence of statistical significance does not negate the diverse and unique experiences faced by caregivers of children with different types of disabilities. Each disability presents its own set of challenges and demands, which can have an impact on caregivers’ well-being and their overall QoL. The lack of significance in our study may be attributed to various factors, such as the sample size, specific characteristics of the participants, or the complexity of the caregiving experience.

In relation to the severity of the disability and the presence of another disabled child in the family, we specifically observed a significant difference in the bodily pain domain of the SF-36. This indicates that caregivers of children with severe disabilities tended to have lower QoL scores compared to those caring for children with milder disabilities. These results are consistent with previous research that has highlighted the physical strain and health issues faced by caregivers of disabled children [30,36,49]. It was observed that the mothers of children with disabilities assigned more importance to health and functioning when compared to the mothers of children without disabilities [10]. The increased physical demands of caregiving, including lifting, assisting with mobility, and managing medical needs, can contribute to higher levels of bodily pain among caregivers [38,40,50]. This is aligned with a previous study, which reported that the parents of children with disabilities such as cerebral palsy experienced physical stress. Specifically, 49.5% of parents reported feeling tired daily, and among them, 48.4% attributed their exhaustion to the demands of caring for their children with cerebral palsy. Consequently, more than half of these caregivers reported having low QoL [36]. Supporting this, other studies conducted in different countries, including the United Kingdom, India, Turkey, and Iran, have also reported a significant decrease in family QoL as the severity of the autism spectrum disorder increased [51-54]. Furthermore, we found that caregivers who had multiple children with disabilities experienced more significant pain-related challenges, which had an impact on their QoL. Consequently, our study revealed that caregivers who are dealing with multiple disabled children may face additional challenges and strains that could potentially impact their perceived health changes. The increased physical, emotional, and financial demands associated with caring for multiple children with disabilities can contribute to significant pain-related challenges and unique burdens reported by caregivers, ultimately affecting their overall QoL. This finding contradicts a previous study that found no significant association between the presence of another child with cerebral palsy in the family and QoL [31]. This underscores the importance of recognizing and addressing the specific needs of caregivers with multiple disabled children through targeted interventions, support systems, and resources that can be aimed at alleviating pain-related issues and improving overall QoL.

Finally, this article has highlighted the interconnectedness of different aspects of QoL among caregivers of disabled children. We found strong positive associations between physical, emotional, and social well-being. This indicates that interventions targeting energy levels and fatigue management can have positive effects on emotional well-being, social functioning, and pain management for caregivers. Likewise, a previous study found that the primary caregivers of children with cerebral palsy had significantly lower scores in the physical functioning, vitality, general health, and emotional role dimensions of the SF-36 subscale when compared to a comparison group [55]. By recognizing these interrelationships, interventions can address multiple aspects of QoL simultaneously, thus improving overall well-being. Healthcare professionals, policymakers, and support organizations should take a holistic approach, considering physical, emotional, and social factors, to develop comprehensive programs that can meet the multifaceted needs of caregivers. Following up with an integrated approach could enhance the QoL of caregivers and improve the care provided to disabled children.

The present study has several notable strengths. First, it encompasses a representative sample of family caregivers from diverse regions of Saudi Arabia, thereby ensuring a comprehensive perspective on the population under investigation. Second, the study incorporates the utilization of the SF-36, a standardized instrument for assessing QoL, which enhances the reliability and validity of these findings. Last, this study addresses an important and under-researched topic that aligns with the goals of Saudi Arabia’s Vision 2030, thereby amplifying its relevance and societal implications. However, it is important to acknowledge certain limitations. The sample size was relatively small, potentially limiting the statistical power to detect significant differences. Furthermore, our reliance on self-reported measures introduced the possibility of response biases and subjective interpretations. Additionally, the study used an online data collection method, which could have introduced selection bias by excluding caregivers without internet access or technological literacy. Moreover, due to the cross-sectional design of this study, causality could not be inferred from these studies. Further longitudinal studies could provide more comprehensive insights into the dynamic nature of caregivers’ QoL over time. It is also worth noting that the study did not account for the leisure activities of caregivers, which could also contribute to their well-being. Last, this study’s results may not be generalizable to other populations outside of Saudi Arabia due to the unique cultural context and support services available for individuals with disabilities in this country.

## Conclusions

In conclusion, our study highlights the QoL experienced by the family caregivers of disabled children in Saudi Arabia. The findings shed light on the impact of sociodemographic factors and child characteristics on various aspects of their health. Variations in QoL were observed among caregivers, indicating significant challenges for some individuals. Factors such as education level, relationship with the child, the severity of the disability, and the presence of multiple disabled children in the family influenced caregivers’ QoL. 

Recommendation

Based on the findings of this study, we recommend that healthcare professionals, policymakers, and support organizations in Saudi Arabia prioritize the development of targeted interventions and support systems that address the diverse needs of caregivers, with a focus on areas such as emotional well-being, fatigue management, and holistic support. Furthermore, conducting longitudinal studies with larger and more diverse samples will deepen our understanding of caregivers' well-being and inform the development of tailored interventions, ultimately improving the QoL of family caregivers and promoting a more inclusive and supportive society. Future research should also consider the recreational activities of caregivers, which may positively impact their overall wellness.
